# Relationship between Mastery and Caregiving Competence in Protecting against Burden, Anxiety and Depression among Caregivers of Frail Older Adults

**DOI:** 10.1007/s12603-018-1098-1

**Published:** 2018-09-12

**Authors:** Ee-Yuee Chan, G. Glass, K.-C. Chua, N. Ali, W.-S. Lim

**Affiliations:** 1 Nursing Service, Tan Tock Seng Hospital, 11 Jan Tan Tock Seng, Singapore, Singapore; 2 Alice Lee Centre of Nursing Studies, National University of Singapore, Singapore, Singapore; 3 Institute of Psychiatry, Psychology and Neuroscience, King's College London, London, UK; 4 Department of Geriatric Medicine, Institute of Geriatric and Active Aging, Tan Tock Seng Hospital, 11 Jan Tan Tock Seng, Singapore, Singapore; 5 Yong Loo Lin School of Medicine, National University of Singapore, Singapore, Singapore; 6 Tan Tock Seng Hospital, 11 Jalan Tan Tock Seng, Nursing Service, Annex 1, 308433, Singapore, Singapore

**Keywords:** Mastery, caregiver competence, carers, caregivers, elderly, older adults, caregiver burden, anxiety, depression, carer stress

## Abstract

**Objective:**

Studies suggest the protective effect of mastery and caregiving competence against psychological stressors of caregiving in the context of dementia, although the interplay between the two with caregiver outcomes is not well understood. This study examines the independent and moderating impact of mastery and caregiving competence on burden, anxiety and depression among caregivers of older adults with frailty-related care needs.

**Design, Setting and Participants:**

This is a cross-sectional study of 274 older adults-family caregiver dyads from a hospital in Singapore. Mean ages of the older adults and their caregivers were 85 and 59 years respectively.

**Measurements:**

We performed hierarchical linear regression models to examine the independent influence of mastery and caregiving competence on caregiver burden, anxiety and depression. We also examined the interaction effect between mastery and caregiving competence for each outcome.

**Results:**

Mastery and caregiving competence were independently negatively associated with caregiver burden, anxiety and depression. Mastery explained more variance than caregiving competence and had a stronger correlation with all outcomes. There was a statistically significant interaction between mastery and caregiving competence for depression (interaction term beta=.14, p<0.01), but not burden and anxiety. High levels of mastery are associated with less depression. particularly among caregivers with below-average levels of caregiving competence. Likewise, high levels of caregiving competence are associated with less depression. particularly among caregivers with below-average levels of mastery.

**Conclusion:**

Our findings suggest potential benefits adressing targeted interventions for mastery and caregiving competence of caregivers to older adults as they independently influence caregiver outcomes and moderate each other's effect on depression. Mastery-based interventions should be incorporated into current caregiver training which traditionally has focused on caregiver competence alone.

## Introduction

By 2050, the number of individuals aged 65 and over is expected to rise to 17% of the global population (1). For many older adults, advancing age is associated with increased frailty and the need for caregivers (2, 3). Frail older adults form the large majority of individuals who require family caregivers in the community (4). Unaddressed demands of care that exceed the ability of family caregivers to cope can lead to stress which can in turn cause caregiver burden, anxiety and depression (5, 6, 7, 8, 9). Caregiver burden has been shown to increase the risk of institutionalization and escalate healthcare costs among persons with dementia (10, 11).

Mastery refers to an individual's perceived global sense of control over life situations and not being fatalistic (12, 13, 14, 15). In contrast, caregiving competence relates to an individual's self-assessment within the domain of caregiving, specifically to the self-appraisal of their adequacy and performance as a caregiver (14, 16). In the stress coping literature, mastery and caregiving competence are malleable constructs of the coping process which emphasize problem-focused and emotionfocused approaches to coping (6, 16, 17, 18, 19). Within the Stress Process Model, caregiver stress is seen as the consequence of a process that involves four interrelated domains: caregiving background and context of stress, stressors, mediators of stress, and outcomes or manifestations of stress (14). In this model, mastery and caregiving competence are situated as intervening resources that potentially could intervene in the stress process to protect against the psychological stresses of caregiving (14, 20).

Earlier studies, mainly in the context of caregivers of persons with dementia, found a negative association between both dimensions of mastery and caregiving competence, and adverse psychological outcomes. Mastery has been shown to have a negative association with depression and physiological responses to stress (13, 20, 21). The literature on caregiver competence also suggested a negative association with depression (16, 19, 22). Importantly, the viability of mastery and caregiving competence as malleable constructs are corroborated by interventional studies that aim to equip caregivers with the requisite knowledge and skills to effectively perform their caregiving role (17, 22, 23, 24). The most pronounced improvement in perceived mastery and caregiving competence was observed amongst caregivers with the lowest levels of mastery or competence and highest levels of burden, suggesting that the most distressed caregivers may benefit most from such interventions (17).

Taken together, this suggests that both mastery and caregiving competence are separate cognitive constructs in the stress process framework that may buffer against the stressors of caregiving through enhancing the problem-focused coping mechanism and responsiveness to experiences and learning opportunities (16, 19). This raises the intriguing possibility that the two constructs of mastery and competence in combination may interact to produce an accentuated protective effect against negative psychological effects of caregiving. To our knowledge, no study has simultaneously examined both mastery and caregiving competence, and their interaction, across the breadth of caregiver outcomes such as depression, anxiety and burden, amongst caregivers of frail older adults. Available studies that examine the impact of mastery and competence on caregiver outcomes are primarily in the context of dementia in Western populations (22, 25). Understanding the interplay between mastery and competence in protecting against adverse caregiving outcomes would allow interventions to be tailored to enable caregivers to thrive as they care for their loved ones.

Therefore, the aim of the current study is to determine the independent effect of mastery and caregiving competence, as well as how they interact with one another, against caregiver burden, anxiety and depression among family caregivers of frail older adults in a multi-ethnic Asian population. These outcomes were chosen as they are sensitive to life situations and are commonly used in studies of stress. We hypothesized that both mastery and caregiving competence will be negatively associated with caregiver burden, anxiety and depression.

## Methods

### Setting

This was a cross-sectional questionnaire survey from a larger longitudinal study on older patient-family caregiver dyads from the acute and subacute geriatric and general medical wards of a 1300-bedded tertiary hospital in Singapore. We defined caregivers as family members who have the responsibility of decision making and caring for older adults with frailty-related care needs. We consecutively recruited adult family caregivers caring for patients who fulfilled the following criteria: a) aged 65 and above, b) dependent in activities of daily living as documented in their clinical notes, c) current hospital admission is non-elective, and d) not resident of assisted living or longterm care facilities. We excluded patients with no identified caregivers, and who are dangerously ill or receiving palliative care.

### Data Collection

Ethics approval was obtained from the Domain-Specific Institutional Review Board of the National Healthcare Group Singapore. Caregivers were asked to respond to the questionnaire based on their situation at home prior to the current hospitalization. The face-to-face survey took approximately 30 to 45 minutes to complete and were administered by trained interviewers who asked caregivers to recall the situation two weeks prior to the current hospitalization.

### Measurements

#### Mastery

We used the 7-item scale developed by Pearlin and Schooler (15) to measure mastery. It has been used to study caregivers caring for patients with varying conditions from cancer to Alzheimer's disease (16, 21, 26, 27, 28). The scale included items such as “I have little control over the things that happen to me.” and “There is little I can do to change many of the important things in my life”. Each item was scored on a four-point scale; total scores ranged from 7 to 28 with higher scores indicating higher levels of mastery. The Cronbach's α was 0.75–0.79 (14, 15).

#### Caregiving Competence

The Caregiving Competence Scale measures self-appraisal of one's efficacy at caregiving (14, 16, 25) and has been widely used in caregiving research (17, 22, 24). Participants responded to statements such as whether they believe that they have learned how to deal with a very difficult situation, and their appraisal of whether they were “a good caregiver”. The total scores of this 4-item scale ranged from 4 to 16, with higher scores indicating higher levels of caregiving competence. It demonstrates good internal consistency (Cronbach's α = .74) (14, 16).

#### Caregiver Burden

The Zarit Burden Interview Scale (ZBI) has 22 items to assess the level of caregiver burden (29). The items were rated on a 5-point scale (0 to 4), with higher scores reflecting higher levels of caregiver burden. Total scores ranged from 0 to 88. The ZBI has good internal consistency with Cronbach's α ranging from 0.87 to 0.93. It has been validated locally among caregivers of persons with dementia (30).

We used the Hospital Anxiety and Depression (HADS) Scale to measure caregiver anxiety and depression. The 14-item scale comprised of two seven-item subscales: anxiety and depression. Each item was rated on a four-point scale with higher scores indicating higher levels of anxiety or depression. Total scores for each subscale ranged from 0 to 21. Both HADS subscales displayed good internal consistency (Cronbach's α = 0.82 for anxiety subscale, and 0.77 for depression subscale) (31).

#### Other Variables

We collected patient and caregiver demographics such as age, gender, educational level, and patient-caregiver relationship. We also collected information on care demands such as living arrangements, presence of domestic helpers, patients' functional status using the Barthel Index (32), and the severity of behavioural symptoms using the Neuropsychiatric Inventory-Questionnaire (NPI-Q) (33).

### Data Analysis

We performed descriptive analysis of the characteristics of caregivers and patients. We examined univariate associations between mastery and caregiving competence with the outcomes of caregiver burden, anxiety and depression.

We conducted separate models of hierarchical multiple linear regression to examine the associations of two predictor variables, mastery and caregiving competence, with three caregiver outcomes (i.e. caregiver burden, anxiety, and depression, respectively). We built the regression models in this sequence for the predictor variables: mastery alone, caregiving competence alone, and mastery and caregiving competence concurrently. The associations were first explored with caregiver burden, followed by HADS anxiety and then HADS depression. We checked the assumptions of normality, linearity, multicollinearity and homoscedasticity.

We built a base model to control for the background influence of background caregiver and care-recipient characteristics, namely, caregiver age, gender, educational level (tertiary education or lower), living arrangements, presence of domestic helpers, neuropsychiatric behavioural symptoms (Neuropsychiatric Inventory-Questionnaire (NPI-Q) severity) (33), and functional independence (Barthel Index) (32). Next, we separately entered mastery and caregiving competence (Models 1a and 1b), noting the R2 change from the base model. Finally, we entered mastery, caregiving competence and their interaction term into the same model (Model 2).

For models with the interaction term, we used centered predictor variables (subtracting the mean from each case) to limit the effects of multicollinearity associated with the use of multiplicative terms. A moderating effect is indicated if the interaction term (centered mastery*centered caregiving competence) is statistically significant. Interaction plots were constructed for each outcome at different levels of mastery or caregiving competence (i.e. centered mean and +/- 1 SD). When the interaction was statistically significant, we examined the interaction plot to see how the association between the outcome and mastery depended on caregiving competence, and conversely, how the association between the outcome and caregiving competence depended on mastery.

## Results

### Characteristics of Caregivers and Care-recipients

A total of 274 patient-caregiver dyads participated in the study (Table 1). Caregivers were mostly older adults (mean age of 59 years old), female (65%), married (61%), children of the care-recipients (71%) with secondary level education or lower (64%). The majority (84.7%) were living with their care-recipients and provided caregiving that exceeded 40 hours/ week. Care-recipients have a mean age of 85 years old, mainly female (64%) and half were diagnosed with dementia (50.4%). Caregivers reported moderate levels of caregiver burden, and low levels of anxiety and depression (Table 1).

**Table 1:** Demographic characteristics of caregivers and care-recipients (N = 274)

**Demographic Characteristics**	**Caregivers’ Characteristics**	**Care-recipients’ Characteristics**
	N (%) / Mean ±SD	N (%) / Mean ±SD
Age, year	59.1 ± 10.5	85.29 ± 8
Female	178 (65.0%)	175 (63.5%)
Married	166 (60.6%)	
Education (< Tertiary)	175 (63.9%)	
Ethnicity		
Chinese	229 (83.6%)	
Malay	20 (7.3%)	
Indian	16 (5.8%)	
Others	9 (3.3%)	
Currently employed (Full/ Part-time)	136 (49.6%)	
Relationship to care-recipient		
Spouse	47 (17.2%)	
Child	194 (70.8%)	
Others	33 (12.0%)	
Living with care-recipient	232 (84.7%)	
Presence of domestic helper	135 (49.3%)	
Years of caregiving		
<5 y	121 (44.2%)	
5 to <10 y	66 (24.1%)	
≥10 y	87 (31.8%)	
Weekly hours of caregiving	88.96 ± 66.13	
Mastery (7 to 28)	19.42 ± 3.29	
Caregiving competence (4 to 16)	11.88 ± 2.32	
Caregiver Burden (ZBI) (0 to 88)	29.38 ± 15.26	
Anxiety (HADS) (0 to 21)	6.79 ± 4.80	
Depression (HADS) (0 to 21)	5.91 ± 4.51	
Dementia diagnosis		138 (50.4%)
Barthel Index scores (10 to 30)		19.48 ± 5.59
NPI-Q Severity (0-36)		7.37 ± 6.59

ZBI refers to Zarit Burden Interview; HADS refers to Hospital Anxiety and Depression Scale; NPI-Q refers to Neuropsychiatric Inventory-Questionnaire

### Mastery and Caregiving Competence Scales

Total mean scores for mastery and caregiving competence were 19.42 (SD = 3.29) and 11.88 (SD = 2.32) respectively. Both scales exhibited good internal consistency (Cronbach's alpha: 0.78 for mastery scale; 0.74 for caregiving competence scale) (Table 2). The items which were most highly endorsed on the mastery scale were “What happens to me in the future mostly depends on me”. [mean (SD) = 3.02 (0.62)] and “I can do just about anything I really set my mind to do”. [mean (SD) = 2.85 (0.63)]. For caregiving competence, the items with the highest means were that they have learned how to deal with a very difficult situation [mean (SD) = 3.19 (0.81)] and their self-confidence in coping with the daily ups and downs as a caregiver [mean (SD) = 3.00 (0.79)].

**Table 2:** Characteristics of personal mastery and caregiving competence scales (N=274)

**Masterya (Cronbach alpha 0.78)**	**Mean ±SD**
1 I have little control over the things that happen to me	2.70 (0.79)
2. There really is no way I can solve some of the problems I have	2.68 (0.74)
3. I can do just about anything I really set my mind to do	2.85 (0.63)
4. Often I feel helpless in dealing with the problems of life	2.72 (0.77)
5. Sometimes I feel like I am being pushed around in life	2.78 (0.74)
6. What happens to me in the future mostly depends on me	3.02 (0.62)
7. There is little I can do to change many of the important things in my life	2.66 (0.70)
**Caregiving Competencea (Cronbach alpha 0.74)**	**Mean ±SD**
1. I have learned how to deal with a very difficult situation	3.19 (0.81)
2. All in all, I feel that I am a good caregiver	2.80 (0.77)
3. Putting all these things (daily ups and downs that I face as a caregiver) together, how competent do I feel	2.89 (0.74)
4. Putting all these things (daily ups and downs that I face as a caregiver) together, how self-confident do I feel	3.00 (0.79)

a. Each item ranged from 1 to 4

Table 3 showed that mastery was moderately correlated with caregiving competence (r = 0.40, p < 0.01). The Caregiver Burden was more strongly correlated with mastery (r = -0.59, p < 0.01) than with caregiving competence (r = -0.32, p < 0.01). Similarly, both caregiver anxiety and depression exhibited moderate correlations with mastery but weaker correlations with caregiving competence (34).

**Table 3:**
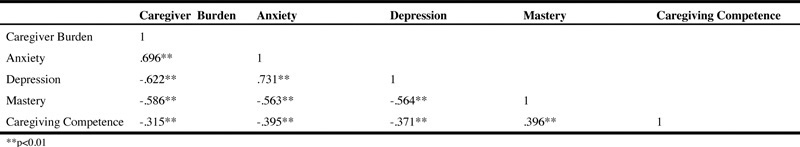
Correlation matrix of model variables (N=274)

### Regression Analysis

Model 1a with mastery as the predictor variable explained 44% of the total variance (F = 27.24, p < 0.001; beta = -0.49, p <0.001) and an additional 21% variance compared with the base model. Model 1b with caregiving competence as the predictor variable explained 30% of the variance (F = 15.54, p < 0.001; beta = -0.28, p < 0.001) and only 7% additional variance compared with the base model. Model 2 with mastery, caregiving competence and their interaction term, explained 45% of the variance (F = 23.02, p < 0.001); however, the interaction term did not explain a reliable amount of additional variance and was not statistically significant (beta = 0.08, p = 0.093).

### Regression Analysis

Caregiver Burden (Table 4, Panel 1)

**Table 4:**
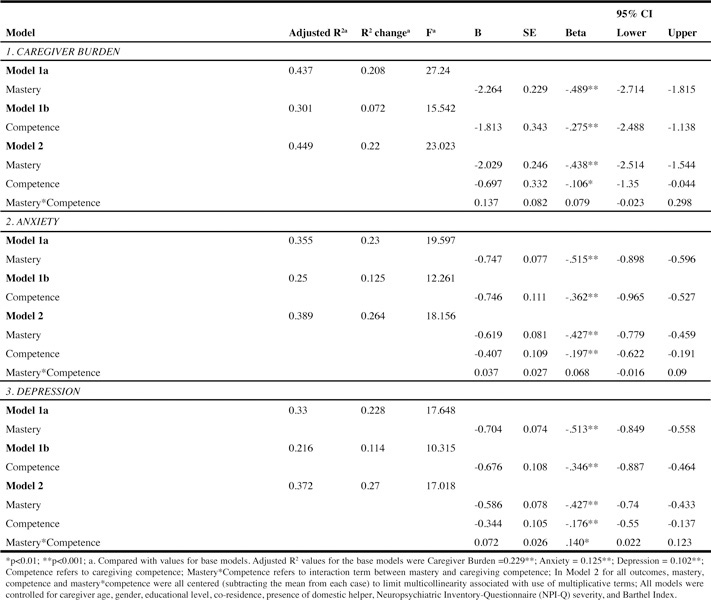
Multiple linear regression for caregiver burden, anxiety and depression (N=274)

Model 1a with mastery as the predictor variable explained 44% of the total variance (F = 27.24, p < 0.001; beta = -0.49, p <0.001) and an additional 21% variance compared with the base model. Model 1b with caregiving competence as the predictor variable explained 30% of the variance (F = 15.54, p < 0.001; beta = -0.28, p < 0.001) and only 7% additional variance compared with the base model. Model 2 with mastery, caregiving competence and their interaction term, explained 45% of the variance (F = 23.02, p < 0.001); however, the interaction term did not explain a reliable amount of additional variance and was not statistically significant (beta = 0.08, p = 0.093).

### Anxiety (Table 4, Panel 2)

Model 1a with mastery as predictor model explained 36% of the total variance (F = 19.60, p <0.001, beta = -0.52, p < 0.001) and 23% additional variance compared, with the base model. On the other hand, the caregiving competence predictor model (Model 1b) explained 25% of total variance (F = 12.26, p < 0.001, beta= -0.36, p < 0.001) and 13% additional variance compared, with the base model. Model 2 explained 39% of the variance (F = 18.16, p < 0.001), with the interaction term not statistically significant (beta = 0.07, p = 0.169).

### Depression (Table 4, Panel 3)

Model 1a with mastery as predictor variable explained 33% of the variance (F = 17.65, p < 0.001, beta = -0.51, p < 0.001) and 23% additional variance compared, to the base model. In comparison, the caregiving competence predictor model (Model 1b) explained 22% total variance (F = 10.32, p < 0.001, beta = -0.35, p < 0.001) and 11% additional variance. Model 2 explained 37% of the total variance with the interaction term (beta=.14, p < 0.01), mastery (beta = -0.43, p < 0.001) and caregiver competence (beta = -0.18, p < 0.01).

### Interaction Plots for Depression

**Figure 1:**
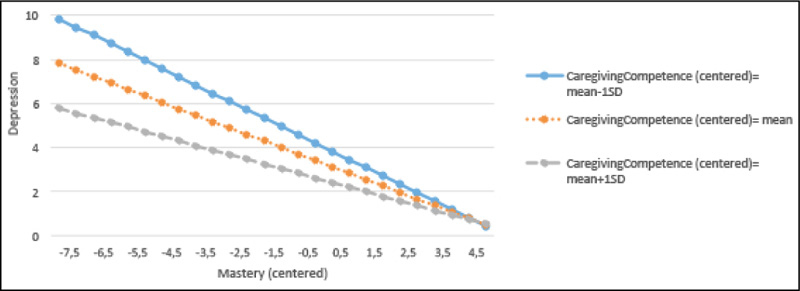
Regression lines for the relations between mastery and depression by the different levels of caregiving competence

**Figure 2:**
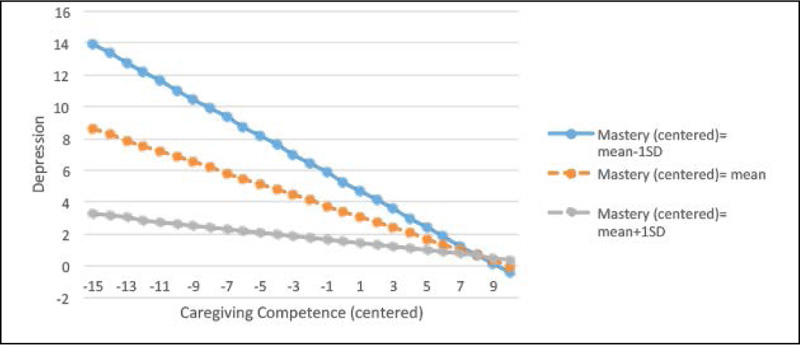
Regression lines for the relations between caregiving competence and depression by the different levels of mastery

As illustrated in Figure 1, caregivers with higher levels of mastery reported lower levels of depression. This relationship is notably stronger among caregivers with lower levels of caregiving competence, compared to those with average or higher levels of caregiving competence. Similarly, in Figure 2, high levels of caregiving competence exert a buffering effect on the mastery-depression relationship. This relationship is also stronger among caregivers with lower levels of mastery, compared to average or higher levels of mastery.

## Discussion

To our best knowledge, this is the first study amongst family caregivers of frail older adults which explored the relationships between both mastery and caregiving competence with caregiver psychological outcomes. Building upon the results of earlier studies that examined the negative effects on caregiver psychological health of either mastery or caregiving competence in isolation (20, 22), we found that independently, mastery and caregiving competence were negatively associated with caregiver burden, anxiety and depression. Compared to caregiving competence, mastery explained a greater proportion of the variance in all regression models and exhibited a stronger correlation with the outcomes of burden, anxiety and depression. In addition, for the outcome of depression, a high level of mastery can mitigate the negative impact of low caregiving competence on depression. Likewise, a high level of caregiving competence can mitigate the negative impact of low mastery on depression.

Our study supports the notion that higher levels of mastery or caregiving competence may buffer against negative psychological outcomes (20, 22). Past studies found that mastery may be a psychological resource in that it buffers against stressors and negative wellbeing among caregivers of individuals with dementia (20, 28). Sub-group analyses of our results showed that mastery and competence exert a similar protective influence for caregivers of older adults with and without dementia. Hence, our study extends the possible protective effect of mastery and caregiving competence to caregivers of frail older adults beyond dementia. Caregivers of frail older adults often face high psychological distress as frail older adults require high care demands and are vulnerable to deterioration in their health status (35). In particular, the mean caregiver burden scores in our sample of hospitalized frail older adults was much higher than that of caregivers for individuals with dementia attending a memory clinic in a previous local study (36). Additionally, despite differences in sociocultural context and care setting, the total mean scores for mastery and caregiving competence in our study were comparable to those reported largely from community studies in the West (16, 17, 24, 37).

Applying the Social Cognitive Theory, individuals with a higher perceived mastery and caregiving competence would be more likely to engage in positive thinking and coping, and problem-solving behaviours in managing their life in general and in caregiving situations. When they perceived that these coping strategies are successful, they would continue utilizing them due to positive reinforcement, facilitating the internalization of positive adaptive strategies and in the process further contributing to a greater sense of mastery and competence (16, 37, 38). This virtuous cycle would consolidate gains from the caregiving experience such as personal fulfilment, satisfaction from helping a loved one, or gaining new caregiving skills, which in turn would further buffer against the negative consequences of caregiving (39, 40).

Critically, our study demonstrated the novel finding of an interaction effect between mastery and caregiving competence on depression. The mechanism through which these two constructs interplay is not fully understood. Nevertheless, this could reflect an accentuation of the problem-focused coping strategies reflected by individuals with high levels of either mastery or caregiving competence (41, 42). Past studies highlighted that problem-focused coping strategies were associated with a lower likelihood of the caregiver expressing depressive symptoms (41, 42). Hence, high perceived levels of either mastery or caregiving competence can accentuate each other's impact to result in greater problem-focused coping strategies, which would mitigate the depressive symptoms associated with the stresses of caregiving.

The findings that mastery plays a more critical role than caregiving competence highlight the support that caregivers would need to manage their other roles in addition to their caregiving role. This is of particular importance to healthcare professionals, since the majority of caregiver training by healthcare professionals has been aimed at increasing competence in caregiving-specific skills and knowledge (24, 43). While this might possibly contribute to an increased sense of competence (24), our findings suggest that strategic strategies that specifically target mastery are also necessary. There may thus be a need to review the concept of caregiver education and engagement by healthcare professionals to incorporate the concept of mastery into caregiver training programmes. p ]Our findings have implications for theory and practice. Since mastery and caregiving competence are potentially malleable (17, 22, 24), it is important that both constructs be considered in the caregiver stress process framework, although mastery may have a more critical role. Traditionally, the focus of healthcare professionals has largely been on equipping caregivers to raise their caregiving competence. Our study highlighted that it is equally important to boost caregiver mastery. Psychoeducational interventions that may increase mastery include skills such as positive cognitive reframing and problem-solving skills, but these were developed mainly in the context of dementia. As hospitalization provides an opportune time to identify caregivers in need of such interventions, in particular the most distressed caregivers who are likely to most benefit (17), it is important to adapt interventions to meet the needs of caregivers across the spectrum to include different cultural groups and non-demented frail older adults.

Some limitations of the study are worth highlighting. Given the cross-sectional design, we are unable to exclude reverse causality between the mastery and caregiving competence constructs, and caregiver outcomes. We await results from our longitudinal study, which would provide more insights into the interplay between mastery and competence, and caregiver outcomes in the Stress Process Model. Future studies are needed to further explore the interaction effect between mastery and caregiving competence on depression and its absence on anxiety and burden. Our findings may have been subjected to recall bias as respondents were asked to recall the situation two weeks prior to admission. Nevertheless, we believe that recall error is low due to the short recall time frame and the salient nature and frequencies of the events (44). Lastly, our results may not be generalizable beyond the study context of caregivers experiencing the stressors associated with hospitalization of their family members in a multi-ethnic Asian context. We propose that future studies employ qualitative methodologies for a more in-depth exploration of the mastery constructs that embrace the respective socio-cultural contexts.

## Conclusion

Our study supports the notion that mastery and caregiving competence may protect caregivers from the negative psychological outcomes of caregiving, with mastery having a greater impact than caregiving competence on caregivers' burden, anxiety and depression. Nevertheless, both constructs are important to consider in view of the interaction effect on depression, such that high mastery and high caregiving competence were associated with lower depression levels. The quiet epidemic of caregivers with low mastery and competence and high burden is a major health concern as it can have a direct toll on the caregivers' health, and wider ramifications including care recipients' health and healthcare service utilization. Our findings suggest the need for assessment and targeted interventions to boost mastery and caregiving competence in at-risk caregivers, as mastery and caregiving competence independently influence caregiver outcomes and moderate each other's effect on depression.
